# Deciphering the anti-angiogenic effect of endostatin/cyclophosphamide to normalize tumor micrangium through notch signaling pathway in colon cancer

**DOI:** 10.1186/s12957-015-0761-9

**Published:** 2016-01-14

**Authors:** Jin-Yan Lv, Tai-Yuan Hu, Ruo-Yu Wang, Jin-Ming Zhu, Gang Wang

**Affiliations:** 1 Department of Oncology, Affiliated Zhongshan Hospital of Dalian University, Jie Fang Street,Zhongshan District, Dalian, Liaoning 116001 Peoples’ Republic of China; 2 Library, Liaoning University of International Business and Economics, Dalian, Liaoning 116001 Peoples’ Republic of China

**Keywords:** Colon cancer, Angiogenesis, Micrangium, Endostatin, Cyclophosphamide

## Abstract

**Background:**

The invasion of colon cancer is associated with the tumor angiogenesis. Endostatin is an important anti-angiogenic agent, and the additive effect of endostatin with a chemotherapeutic agent, cyclophosphamide, on micrangium has not been established.

**Methods:**

Male BALB/c strain nude mice were injected with human colorectal carcinoma cells (HCT-116). The mice were divided into four groups (*n* = 15, each group) and were treated with different concentrations of endostatin (15, 10, and 5 mg/kg/day), cyclophosphamide (20, 10, and 5 mg/kg/day), and combination of endostatin/cyclophosphamide (15 + 20, 15 + 10, and 15 + 5 mg/kg/day). The tumor inhibition rate was evaluated, followed by the quantification of messenger ribonucleic acid (mRNA) and protein expression of notch signaling components NOTCH-1, NOTCH-3, NOTCH-4, JAG-1, DLL-4, Hes-1, and Hey-1 using quantitative polymerase chain reaction (qPCR). The protein expression of NOTCH-3, JAG-1, and DLL-4 was confirmed using western blotting. Microvessel density (MVD) was evaluated to detect micrangium following the treatment.

**Results:**

The endostatin/cyclophosphamide-treated samples exhibited an additive effect on the tumor inhibition rate and the microvessel count. NOTCH-1, NOTCH-3, NOTCH-4, JAG-1, Hes-1, and Hey-1 expression levels were highly correlated and downregulated in the treated samples, whereas DLL-4 expression was upregulated that accounted for its anti-angiogenic property.

**Conclusions:**

The combination treatment of colon cancer with endostatin and a chemotherapeutic agent, cyclophosphamide proves to be an efficient therapeutic strategy to inhibit the rapid vasculature formation confirmed by the differential expression of notch signaling components.

## Background

Colon cancer forms the third most prevalent cancer in the world, and its progression is critically dependant on the angiogenesis [1]. Angiogenesis forms an important factor in the rapid expansion of tumors by promoting the process of metastasis [2, 3]. The anti-angiogenesis therapy to culminate cancer has now become one of the potential fields of study [4]. Endostatin, an angiogenesis inhibitor, is a naturally occurring cleaved product of carboxyl-terminal of collagen XVIII [5]. Endostatin is reported to suppress the angiogenesis and endothelial cell proliferation, thereby inhibiting the angiogenic proliferation of tumor cells [5]. However, the clinical trials have produced mixed results on the effect of endostatin in the tumor growth [6–9]. Endostatin exhibits anti-tumor property and apoptosis in tumor cells, but some of the independent studies have revealed that endostatin has no direct cytotoxic effect on the tumor cells [6, 10–12]. The combination of endostatin with a chemotherapeutic agent reportedly enhances the efficacy of the tumor inhibition with the reduced side effects [13, 14].

The notch signaling pathway plays a pivotal role in the tumor angiogenesis by promoting progenitor/stem cell proliferation [15]. Notch signaling functions with the combined action of a ligand receptor activity [16]. There are four main kinds of notch receptors (NOTCH 1–4) and five types of ligands (Delta-like ligands (DLL) 1, 3, 4 and Jagged (JAG) 1 and 2). The activation of the notch signaling induces a conformational change by binding of ligands to the notch receptors, which activates the *γ*-secretase protein complex [16, 17]. Notch receptors are cleaved with the activated *γ*-secretase to release the active notch intracellular domain (NICD) which is translocated from the nucleus to bind to the transcriptional factors and recruits co-activators. These co-activators induce the notch downstream target gene expression (Hes-1) that finally activates the notch signaling pathway [18]. The notch signaling pathway is aberrantly activated during colon carcinogenesis and has been reported to be essential in maintaining the development of the intestinal cells [19].

In the present study, we examined the expression of notch signaling receptor NOTCH-1, NOTCH-3, NOTCH-4, ligands JAG1 and DLL-4 along with the downstream targets Hes-1 and Hey-1 to determine the efficacy of a combined therapeutic strategy with endostatin/cyclophosphamide (CTX) to normalize the tumor micrangium associated with colon cancer. The synergy between endostatin and cyclophosphamide is believed to overcome the adverse effects of a chemotherapeutic agent and increase the activity of endostatin that is confirmed by the overall expression of notch signaling receptors and ligands.

## Methods

### Animals

The research involving animals was conducted according to the relevant guidelines and was approved by the local ethical committee. Male BALB/c nude mice (*n* = 70) weighing 20~28.7 g were purchased from the SLAC Company (Shanghai, China). They were caged under optimum conditions (12 h light/dark cycle, temperature of 22 ± 0.5 °C and humidity of 50 ± 10 %), were provided with an appropriate diet, and were under constant inspection for any illness.

### Culture of human colorectal carcinoma HCT-116 cells, SW-480, and SW-620

An endotoxin-free RPMI 1640 (Gibco) supplemented with 10 % FBS (Gibco) and penicillin-streptomycin (Life Technologies) was used to culture human colorectal carcinoma cell lines (HCT-116 cells, SW-480, and SW-620) (ATCC) and were passaged. 3 × 10^5^ cells were cultured for 12 h in a 25 cm^2^ T-flask and maintained in the above conditions to prepare a conditioned medium for cells.

### Development of a colon cancer model and endostatin/CTX treatment

3 × 10^5^ human colorectal carcinoma cells suspended in 0.1 ml of HBSS (Gibco) were injected into the subsplenic capsule of the anesthetized BALB/c strain nude mice. After recovery from the surgery, mice were divided into four random groups (*n* = 15, each group). The group 1 acted as a control and was treated with phosphate buffer saline (PBS) (Gibco). Group 2 was injected with rh-endostatin (Biovision) alone at three different concentrations (15, 10, and 5 mg/kg/day), group 3 with CTX (Sigma-Aldrich) alone (20, 10, and 5 mg/kg/day), and group 4 with the optimized combinations of rh-endostatin + CTX (15 + 20, 15 + 10, and 15 + 5 mg/kg/day). Ten mice (*n* = 5 each) were injected with SW480 and SW620 cells to determine the effect of E + CTX treatment with respect to microsatellite stable colon cancer cells since HCT-116 is microsatellite instability-high (MSI-H). Following 20 days of treatment, the mice were sacrificed and the tumor tissue was then processed for histological examinations. The tumor tissue section (4 μm thick) was formaldehyde-fixed and stained with H&E. The inhibition rate of tumor growth was evaluated using the formula, [(tumor volume of control − tumor volume of treatment) / tumor volume of control]×100 % [15].

### mRNA expression analysis using qPCR

Total RNA was extracted and converted to cDNA. Quantitative polymerase chain reaction (qPCR) (Applied Biosystems, Carlsbad, CA, USA) was performed to assess the relative messenger ribonucleic acid (mRNA) expression levels of NOTCH-1, NOTCH-3, NOTCH-4, JAG-1, DLL-4, Hes-1, and Hey-1 following the endostatin/CTX treatment. Primer probes were acquired commercially from Applied Biosystems. Actin was used as an internal control to normalize the mean Ct values.

### SDS PAGE and western blot

Tissue lysates were subjected to sodium dodecyl sulphate polyacrylamide gel electrophoresis (SDS PAGE) according to Laemmli [20]. Fifty microgram of protein was separated and blotted to a nitrocellulose membrane. The blot was blocked using 5 % blocking solution (nonfat dry milk in PBS) and then incubated with primary antibody to NOTCH-3 (1:200; Santa Cruz, Cat. No. sc-5593), JAG1 (1:500; Abcam, Cat. No. ab7771), DLL-4 (1:500; Abcam, Cat. No. ab7280), and anti-mouse β-actin antibody (1:500; Santa Cruz, Cat. No. sc-4778). The blots were then washed thrice with PBS/0.1 % Tween followed by the incubation of blot in the secondary antibody which was peroxidase-conjugated goat anti-rat IgG (1:5000; Santa Cruz, Cat. No. sc-2032). The blots were washed, and the proteins were detected using a chemiluminescence system. The protein bands were visualized using ImageJ software and quantified by the representation of protein band densities as ratios, with respect to the internal control protein band density.

### Detection of micrangium by counting microvessel density (MVD)

The tumor sections (5 μm) from each group of treated mice were obtained, and immunohistochemical staining was done using nestin marker labeled with streptavidin peroxidase (Invitrogen, USA) as described by Bottini A et al. [21]. The stained microvessels in the tumor were counted at ×400 magnification with the field size of 0.43 mm^2^. The individual vessels were counted using ImageJ software. The average count of five fields per tumor section in the areas with the largest number of microvessels was considered for the analysis. The MVD was calculated by dividing the mean microvessel count by the microscopic field area of magnification [17].

### Statistical analysis

Values are represented as means ± standard errors of the means. One-way analysis of variance (ANOVA) was used for the comparison of the variables, and the statistical significance was set at *p* < 0.05 vs. controls using SPSS 15.0 software. One-way ANOVA was followed up by post hoc tests for *p* < 0.05.

## Results

### Effect of endostatin/CTX on tumor growth and evaluation of tumor inhibition rate

**Fig. 1:**
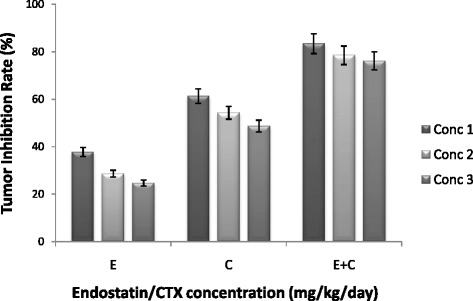
Tumor inhibition rate following endostatin/CTX treatment. The graph represents tumor inhibition rate at different concentrations of endostatin (E), CTX (C), and endostatin + CTX (E + C). The legends *Conc 1*, *Conc 2*, and *Conc 3* represent the treatment concentrations of E, C, and E + C. E was treated at 15, 10, and 5 mg/kg/day, CTX at 20, 10, and 5 mg/kg/day, and E + C at 15 + 20, 15 + 10, and 15 + 5 mg/kg/day. The highest inhibition rate (83.4 %) was exhibited by E + C at 15 + 20 mg/kg/day. One-way ANOVA was performed, and the values were significant with *p* < 0.05 vs. controls

HCT-116-infused colorectal carcinoma mouse model was employed to determine the effect of endostatin/CTX on the tumor growth inhibition. The tumor inhibition rate of the samples treated with endostatin alone was evaluated to be 37.8, 28.6, and 24.7 % for the concentrations 15, 10, and 5 mg/kg/day, respectively. The CTX-treated group 2 showed a comparatively high inhibition rate owing to its chemotherapeutic property, i.e., 61.3, 54.3, and 48.7 % for the concentrations 20, 10, and 5 mg/kg/day, respectively. An optimized concentration for the endostatin + CTX treatment was obtained by selecting 15 mg/kg/day (highest inhibition rate) of endostatin in combination with the different concentrations of CTX. Endostatin + CTX exhibited highest tumor inhibition rate (*p* < 0.05 vs. controls). The inhibition rate was found to be 83.4, 78.5, and 76.2 % for 15 + 20, 15 + 10, and 15 + 5 mg/kg/day, respectively (Fig. 1).

### Endostatin/CTX interferes with the angiogenic function of notch receptor and ligand expression

**Fig. 2:**
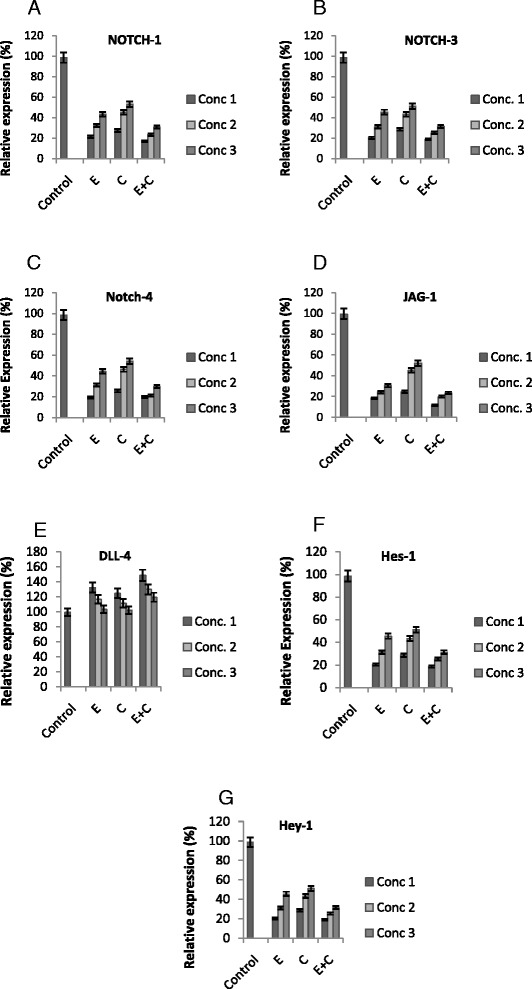
Effect of endostatin/cyclophosphamide treatment on the mRNA expression of NOTCH-3, JAG-1, and DLL-4. The graphs represent relative mRNA expression levels of notch receptors, ligands, and downstream target genes at different concentrations of endostatin (E), CTX (C), and endostatin + CTX (E + C). The legends *Conc. 1*, *Conc. 2*, and *Conc. 3* represent the treatment concentrations of E, C, and E + C. E was treated at 15, 10, and 5 mg/kg/day, CTX at 20, 10, and 5 mg/kg/day, and E + C at 15 + 20, 15 + 10, and 15 + 5 mg/kg/day. **a** NOTCH-1 mRNA expression level. **b** NOTCH-3. **c** NOTCH-4. **d** JAG-1 mRNA expression rate is similar to notch receptor expression with a greater expression in C-treated samples and a lower expression in the E and E + C-treated samples. **e** DLL-4 mRNA expression levels were high in E and E + C-treated samples whereas low in the C-treated samples. **f** Hes-1 mRNA expression. **g** Hey-1 mRNA expression. One-way ANOVA was performed, and the values were significant with *p* < 0.05 vs. control

mRNA expression levels of NOTCH-1, NOTCH-3, NOTCH-4, JAG-1, DLL-4, Hes-1, and Hey-1 were quantified to elucidate the action of endostatin/CTX on the notch signaling pathway in colon cancer. It was found that DLL-4 showed a significantly higher expression (Fig. 2c) compared to the other notch signaling components (*p* < 0.05 vs. controls) (Fig. 2a–d, f, g). The expression was quantified for endostatin/CTX treatment concentrations, and the relative expression levels are shown in Fig. 2. It was inferred that notch receptors and ligands with the downstream targets were expressed positively towards the anti-angiogenic function of endostatin/CTX.

### Notch signaling protein expression confirms the anti-angiogenic activity of endostatin/CTX in colon cancer

**Fig. 3:**
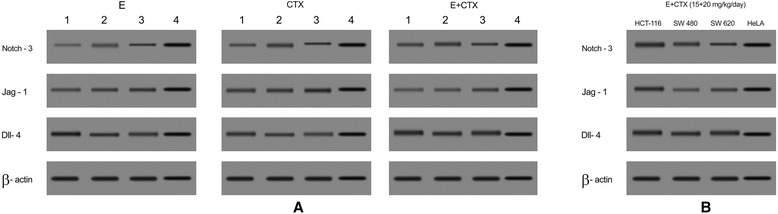
Effect of endostatin/CTX on the notch signaling protein expression. The figure shows the western blot analysis of notch signaling proteins NOTCH-3, JAG-1, and DLL-4 following endostatin/CTX treatment. *E* represents endostatin and *CTX* represents cyclophosphamide. The four lanes represent three treatment concentrations (E at 15, 10, and 5 mg/kg/day, CTX at 20, 10, and 5 mg/kg/day and E + CTX at 15 + 20, 15 + 10, and 15 + 5 mg/kg/day) plus control. β-actin acts as the internal control. **a** NOTCH-3, JAG-1, and DLL-4 expression in E, C, E + C-treated samples. **b** Notch signaling protein expression in different colorectal cell lines (HCT-116, SW-480, SW-620)

The expression of notch signaling proteins, NOTCH-3, JAG-1, and DLL-4 was confirmed using western blot analysis. The Fig. 3 shows expression of notch signaling proteins NOTCH-3, JAG-1, and DLL-4 in different concentrations of endostatin/CTX-treated samples. NOTCH-3 and JAG-1 expression was low, and DLL-4 exhibited a prominent expression in the treated samples. The quantified protein densities showed that endostatin + CTX-treated samples caused the greatest DLL-4 expression. The expression of these notch signaling proteins in different colorectal cell lines (HCT-116, SW480, SW620) was confirmed for the combination treatment (E + CTX). HeLa cell line was used as a control.

### Change in micrangium density in colon cancer-induced mice

**Fig. 4:**
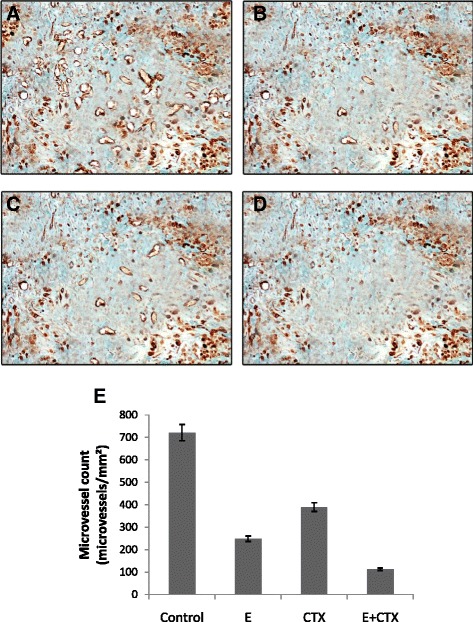
Nestin positive microvessels. **a** Controls with 721 microvessels/mm^2^. **b** Endostatin treated with 248 microvessels/mm^2^. **c** CTX treated with 389 microvessels/mm^2^. **d** Endostatin + CTX treated with 112 microvessels/mm^2^. **e** Bar chart representing average microvessel counts/mm^2^, *p* < 0.05 vs. control. Values are represented as mean + SD

Differentially treated groups of colon cancer-induced mice were counted for the microvessels. Combination of endostatin and CTX (15 + 20 mg/kg/day) showed an additive therapeutic effect with a significantly low microvessel count and a low tumor metastatic rate (Fig. 4d). There was a significant reduction in the microvessel count of the endostatin-treated (15 mg/kg/day) group (Fig. 4b). CTX-treated group (20 mg/kg/day) showed a slight change in the microvessel count (Fig. 4c) but was not as effective as the endostatin-treated group. The control group treated with saline showed a greater microvessel count indicating a high rate of tumor angiogenesis (Fig. 4a). We obtained a mean MVD of 721 microvessels/mm^2^ in group 1 (control), 248 microvessels/mm^2^ in group 2 (endostatin treated), 389 microvessels/mm^2^ in group 3 (CTX treated), and 112 microvessels/mm^2^ in group 4 (endostatin + CTX treated) (Fig. 4e).

## Discussion

Vascularization of cancer cells promotes tumor growth and metastasis [22, 23]. Angiogenesis is important in the progression of cancer and higher the microvessel count, greater the metastatic rate of cancer [24–27]. Vascular endothelial growth factor (VEGF) is a largely studied angiogenesis regulating growth factor that is overexpressed in a majority of solid tumors including colon cancer and is considered to be one of the potential targets [28–30]. However, the development of anti-angiogenic therapy against tumor depends on several other factors. Notch signaling pathway is one of the important regulators of angiogenesis in tumors and is active in colon cancer [15]. Despite an extensive research, the role of notch signaling components is controversial in its function towards the tumor angiogenesis [31, 32]. The notch receptors and ligands are differentially expressed on activation of the notch signaling pathway and contribute individually towards the physiological and pathological conditions [33–35].

Endostatin inhibits angiogenesis, and ample reports prove the efficiency of endostatin as an anti-angiogenic agent in cancer cell progression [4–7, 36]. Endostatin produces additive effects with the conventional chemotherapy agents, thereby reducing the adverse effects caused by these agents [13, 14, 37]. However, the efficiency of the combined therapy could be deteriorated due to the individual or combined toxicities [11, 38]. The tumor cells sometimes become resistant to chemotherapy due to the changing genome, but endostatin has a cytostatic effect and is less susceptible to the drug resistance, thus making the combination therapy more effective [38, 39].

In this study, we demonstrated the effect of endostatin/CTX in the colon cancer inhibition and anti-angiogenesis through notch signaling pathway. The combined therapy of endostatin/CTX significantly inhibited the growth of tumor (Fig. 1) and reduced the microvessel count (Fig. 4d). The endostatin treatment did not have a direct effect on tumor growth (Fig. 1) but reduced the microvessel count owing to its anti-angiogenic property (Fig. 4b). The CTX-treated group showed a greater tumor inhibition rate (Fig. 1) than the endostatin-treated group, and the microvessel counts were greater (Fig. 4c). These observations show that endostatin/CTX combination therapy is effective in inhibition of tumor growth as well as in the inhibition of neovascularization. The combination therapy brings together the individual effect of endostatin as an anti-angiogenic agent and the effect of CTX towards the inhibition of tumor growth. However, CTX has shown positive result as an anti-angiogenic chemotherapeutic agent [40, 44] and hence to further establish the anti-angiogenic property of endostatin/CTX, the mRNA and protein expression of notch signaling components (NOTCH-3, JAG-1, and DLL-4) were studied.

Reports indicate that NOTCH-3, JAG-1, and DLL-4 are among the most altered notch signaling genes in cancer [40]. The expression of NOTCH-3 and JAG-1 is highly correlated and found to be upregulated in cancer. DLL-4 inhibition promotes the formation of new blood vessels that establishes the role of DLL-4 against tumor angiogenesis [33–35, 41]. We quantified the expression levels of these notch signaling components to confirm the function of endostatin/CTX in the colon cancer angiogenesis. The endostatin/CTX (E + C) combination therapy (15 + 20 mg/kg/day) downregulated the NOTCH-3 and JAG-1 whereas upregulated the DLL-4 mRNA and protein expression (Fig. 3). The endostatin (E) and CTX (C)-treated samples exhibited almost similar expression levels for NOTCH-3, JAG-1, and DLL-4 that confirms the additive effect of the combination therapy against tumor angiogenesis. The observations also confirm that the endostatin and CTX alone inhibit the neovascularization through the notch signaling pathway. The upregulation of DLL-4 in the treated samples confirms its anti-angiogenic ability which is in compliance with the previous reports [35, 41, 42] and resolves the contradictions relating notch signaling pathway and its effect in tumor angiogenesis.

The MVD was performed for the concentrations of endostatin and CTX that provided the maximum tumor inhibition rate (Fig. 4). There is only a difference of 136 microvessels/mm^2^ between the endostatin-treated and endostatin + CTX-treated samples, whereas the CTX-treated samples exhibited a greater microvessel count (Fig. 4a, d). These variations indicate that CTX is not primarily involved in anti-angiogenesis but its combination with the endostatin enhances the effect drastically. MVD was performed using nestin marker to detect the micrangium. Nestin has been particularly reported as an efficient marker to detect the presence of newly formed micrangium in colorectal cancer [43]. Further study is essential to elucidate the mechanism of CTX on the colon cancer angiogenesis. The activity of endostatin with the other potent chemotherapeutic agents might also open novel prospects in the development of cancer therapy.

## Conclusions

To conclude, endostatin combined with the cyclophosphamide was found to be drastically effective in reducing the microvessel count and significantly increased the tumor inhibition rate in colon cancer. The anti-angiogenic property of the endostatin/CTX was confirmed with the expression of notch signaling component. This therapeutic strategy might prove to be a beacon of hope to curb the tumor angiogenesis.

## Abbreviations

CTX: cyclophosphamide
FBS: fetal bovine serum
HBSS: Hank’s balanced salt solution
MVD: microvessel density
PBS: phosphate buffer saline
SDS PAGE: sodium dodecyl sulphate polyacrylamide gel electrophoresis

## References

[1] Sun W (2012). Angiogenesis in metastatic colorectal cancer and the benefits of targeted therapy. J Hematol Oncol.

[2] Folkman J (2002). Role of angiogenesis in tumor growth and metastasis. Semin Oncol.

[3] Zetter BR (1998). Angiogenesis and tumor metastasis. Annu Rev Med.

[4] Celik I, Sürücü O, Dietz C, Heymach JV, Force J, Höschele I (2005). Therapeutic efficacy of endostatin exhibits a biphasic dose-response curve. Cancer Res.

[5] O’Reilly MS, Boehm T, Shing Y, Fukai N, Vasios G, Lane WS (1997). Endostatin: an endogenous inhibitor of angiogenesis and tumor growth. Cell.

[6] Mundhenke C, Thomas JP, Wilding G, Lee FT, Kelzc F, Chappell R (2001). Tissue examination to monitor antiangiogenic therapy: a phase I clinical trial with endostatin. Clin Cancer Res.

[7] Eder JP, Supko JG, Clark JW, Puchalski TA, Garcia-Carbonero R, Ryan DP (2002). Phase I clinical trial of recombinant human endostatin administered as a short intravenous infusion repeated daily. J Clin Oncol.

[8] Herbst RS, Mullani NA, Davis DW, Hess KR, McConkey DJ, Charnsangavej C (2002). Development of biologic markers of response and assessment of antiangiogenic activity in a clinical trial of human recombinant endostatin. J Clin Oncol.

[9] Kulke MH, Bergsland EK, Ryan DP, Enzinger PC, Lynch TJ, Zhu AX (2006). Phase II study of recombinant human endostatin in patients with advanced neuroendocrine tumors. J Clin Oncol.

[10] Sun L, Ye HY, Zhang YH, Guan YS, Wu H (2007). Epidermal growth factor receptor antibody plus recombinant human endostatin in treatment of hepatic metastases after remnant gastric cancer resection. World J Gastroenterol.

[11] Plum SM, Hanson AD, Volker KM, Vu HA, Sim BK, Fogler WE (2003). Synergistic activity of recombinant human endostatin in combination with adriamycin: analysis of in vitro activity on endothelial cells and in vivo tumor progression in an orthotopic murine mammary carcinoma model. Clin Cancer Res.

[12] Roy Choudhury S, Karmakar S, Banik NL, Ray SK (2012). Targeting angiogenesis for controlling neuroblastoma. J Oncol.

[13] Ma J, Waxman DJ (2008). Combination of antiangiogenesis with chemotherapy for more effective cancer treatment. Mol Cancer Ther.

[14] Cabebe E, Wakelee H (2007). Role of anti-angiogenesis agents in treating NSCLC: focus on bevacizumab and VEGFR tyrosine kinase inhibitors. Curr Treat Options Oncol.

[15] Qiao L, Wong BC (2009). Role of Notch signaling in colorectal cancer. Carcinogenesis.

[16] Meng RD, Shelton CC, Li YM, Qin LX, Notterman D, Paty PB (2009). Gamma-secretase inhibitors abrogate oxaliplatin-induced activation of the Notch-1 signaling pathway in colon cancer cells resulting in enhanced chemosensitivity. Cancer Res.

[17] Lai EC (2004). Notch signaling: control of cell communication and cell fate. Development.

[18] Blaumueller CM, Qi H, Zagouras P, Artavanis-Tsakonas S (1997). Intracellular cleavage of Notch leads to a heterodimeric receptor on the plasma membrane. Cell.

[19] Radtke F, Clevers H, Riccio O (2006). From gut homeostasis to cancer. Curr Mol Med.

[20] Laemmli UK (1970). Cleavage of structural proteins during the assembly of the head of bacteriophage T4. Nature.

[21] Bottini A, Berruti A, Bersiga A, Brizzi MP, Brunelli A, Gorzegno G (2000). p53 but not bcl-2 immunostaining is predictive of poor clinical complete response to primary chemotherapy in breast cancer patients. Clin Cancer Res.

[22] Saphir A (1997). Angiogenesis: the unifying concept in cancer?. J Natl Cancer Inst.

[23] Hagedorn M, Bikfalvi A (2000). Target molecules for anti-angiogenic therapy: from basic research to clinical trials. Crit Rev Oncol Hematol.

[24] Harris AL (1998). Anti-angiogenesis therapy and strategies for integrating it with adjuvant therapy. Recent Results Cancer Res.

[25] Brem H, Folkman J (1975). Inhibition of tumor angiogenesis mediated by cartilage. J Exp Med.

[26] Weidner N, Carroll PR, Flax J, Blumenfeld W, Folkman J (1993). Tumor angiogenesis correlates with metastasis in invasive prostate carcinoma. Am J Pathol.

[27] O’Reilly MS, Holmgren L, Shing Y, Chen C, Rosenthal RA, Cao Y (1994). Angiostatin: a circulating endothelial cell inhibitor that suppresses angiogenesis and tumor growth. Cold Spring Harb Symp Quant Biol.

[28] Achen MG, Stacker SA (1998). The vascular endothelial growth factor family; proteins which guide the development of the vasculature. Int J Exp Pathol.

[29] Takahashi Y, Kitadai Y, Bucana CD, Cleary KR, Ellis LM (1995). Expression of vascular endothelial growth factor and its receptor, KDR, correlates with vascularity, metastasis, and proliferation of human colon cancer. Cancer Res.

[30] Bendardaf R, Buhmeida A, Hilska M, Laato M, Syrjänen S, Syrjänen K (2008). VEGF-1 expression in colorectal cancer is associated with disease localization, stage, and long-term disease-specific survival. Anticancer Res.

[31] Uyttendaele H, Ho J, Rossant J, Kitajewski J (2001). Vascular patterning defects associated with expression of activated Notch4 in embryonic endothelium. Proc Natl Acad Sci U S A.

[32] Parsons DW, Wang TL, Samuels Y, Bardelli A, Cummins JM, DeLong L (2005). Colorectal cancer: mutations in a signaling pathway. Nature.

[33] Zhang Y, Wang Z, Ahmed F, Banerjee S, Li Y, Sarkar FH (2006). Down-regulation of Jagged-1 induces cell growth inhibition and S phase arrest in prostate cancer cells. Int J Cancer.

[34] Konishi J, Kawaguchi KS, Vo H, Haruki N, Gonzalez A, Carbone DP (2007). Gamma-Secretase inhibitor prevents Notch3 activation and reduces proliferation in human lung cancers. Cancer Res.

[35] Li JL, Sainson RC, Shi W, Leek R, Harrington LS, Preusser M (2007). Delta-like 4 Notch ligand regulates tumor angiogenesis, improves tumor vascular function, and promotes tumor growth in vivo. Cancer Res.

[36] O’Reilly MS, Holmgren L, Chen C, Folkman J (1996). Angiostatin induces and sustains dormancy of human primary tumors in mice. Nat Med.

[37] Boehm T, O’Reilly MS, Keough K, Shiloach J, Shapiro R, Folkman J (1998). Zinc- binding of endostatin is essential for its antiangiogenic activity. Biochem Biophys Res Commun.

[38] Brandwijk RJ, Dings RP, van der Linden E, Mayo KH, Thijssen VL, Griffioen AW (2006). Anti-angiogenesis and anti-tumor activity of recombinant anginex. Biochem Biophys Res Commun.

[39] Prokopiou EM, Ryder SA, Walsh JJ (2013). Tumour vasculature targeting agents in hybrid/conjugate drugs. Angiogenesis.

[40] Man S, Bocci G, Francia G, Green SK, Jothy S, Hanahan D (2002). Antitumor effects in mice of low-dose (metronomic) cyclophosphamide administered continuously through the drinking water. Cancer Res.

[41] Hu W, Liu T, Ivan C, Sun Y, Huang J, Mangala LS (2014). Notch3 pathway alterations in ovarian cancer. Cancer Res.

[42] Noguera-Troise I, Daly C, Papadopoulos NJ, Coetzee S, Boland P, Gale NW (2006). Blockade of Dll4 inhibits tumour growth by promoting non-productive angiogenesis. Nature.

[43] Matsuda Y, Hagio M, Ishiwata T (2013). Nestin: a novel angiogenesis marker and possible target for tumor angiogenesis. World J Gastroenterol.

[44] Ren Z, Wang Y, Jiang W, Dai W, Jiang Y (2014). Anti-tumor effect of a novel soluble recombinant human endostatin: administered as a single agent or in combination with chemotherapy agents in mouse tumor models. PLoS One.

